# Physical beneficiation of heavy minerals – Part 2: A state of the art literature review on magnetic and electrostatic concentration techniques

**DOI:** 10.1016/j.heliyon.2024.e32201

**Published:** 2024-05-31

**Authors:** Nnaemeka Stanislaus Nzeh, Patricia Abimbola Popoola

**Affiliations:** Tshwane University of Technology, Pretoria West 0183, South Africa, Chemical, Metallurgical and Materials Engineering Department

**Keywords:** Heavy minerals, Beneficiation, Magnetic, Electrostatic, Concentration, Separation

## Abstract

Recent advancements in the applications of heavy minerals by modern science, engineering, technological and metallurgical industries especially in the demand by nuclear and power industries have significantly increased over the decades. This is the reason for the criticality and commerciality of products of heavy minerals and also necessitated their high demand by industries. The recovery of heavy minerals, such as: Zr and Ti associated minerals from their deposits is dependent on extractive metallurgy of transition and refractory metals from complex minerals. Based on the effectiveness and efficiency of mineral concentration as well as metal extraction, several challenges have been encountered in their recovery process, especially in their separation from associated mineral impurities or gangue. This review is however focused on investigating magnetic and electrostatic physical processing techniques and their applications in the beneficiation and recovery of heavy minerals. This will therefore, serve as a tool in reducing process steps and extraction complexity involved in downstream measures of dissolution and hydrometallurgical processes of the minerals.

## 1 Introduction

It is of general knowledge that the importance and use of heavy minerals (HMs) cannot be over-emphasized. Over the years, interests have emerged in the upgrade and concentration of HMs, as well as their industrial applications. These HMs, such as: ilmenite [TiFeO_3_], rutile [TiO_2_], and zircon [(Zr,Hf)SiO_4_], are very essential sources of titanium (Ti), titanium dioxide, and zirconium (Zr), respectively. HMs do not have specific standard industrial and scientific definitions. However, they are referred to as high density accessory siliclastic sediment mineral constituents. The minerals have high specific gravities (SGs) higher than that of the major mineral constituent framework. They also have greater densities than that of quartz with approximately 2.65 g/cm^3^. For example, most HMs, such as siliclastic grains, are somewhat dense and possess average SGs greater than 2.9. As a result, the minerals with densities ≥3.0 g/cm^3^ are referred to as HMs [[1], [2], [3], [4], [5]]. Based on this premise, and with respect to their high density and SG properties, these minerals are usually referred to as value heavy minerals (VHMs) [1]. Albeit the aforementioned VHMs, other HMs still exist, that may not necessarily be referred to as VHM, as this is often dependent on the economic circumstances surrounding the extractability and consumption of the HMs. These HMs may therefore include: columbite [Fe,Mn,Mg(Nb,Ta)_2_O_6_], tantalite [Fe,Mn,Mg(Ta,Nb)_2_O_6_], kyanite [Al_2_O(SiO_4_)], staurolite [${Fe}_{2}^{2+}$ Al_9_Si_4_O_23_(OH)], andalusite [Al_2_SiO_5_], sillimanite [Al_2_O(SiO_4_)], tourmaline [(Li,Al,Mn,Mg,Fe)_3_(Cr,V,Fe,Al)_6_ (BO_3_)_3_(Al,Si,B)_6_O_18_(F,OH)_4_], scheelite [Ca(WO_4_)], wolframite [(Fe,Mn)WO_4_], xenotime [(Y,Yb)(PO_4_)], cassiterite [SnO_2_], corundum [Al_2_O_3_], monazite [(Ce,Nd,La,Th,Y)PO_4_], magnetite [Fe_3_O_4_], garnet [(U_3_V_2_Si_3_O_12_); where: U is usually Fe,Ca,Mn,Mg and V is usually Al,Cr,Fe^3+^], silica sand [SiO_2_], chromite [(Fe,Mg)(Cr,Al)_2_O_4_], diamonds [C], platinum [Pt] and gold [Au] [1,2,6,7].

The upgrade and process of HM concentrates as well as other low grade minerals up to technical and marketable grades may however involve the employment of basic physical beneficiation process steps or routes, such as magnetic, electrostatic, conventional and enhanced gravity or centrifugal separations prior to froth flotation process and/or downstream pyro-hydrometallurgical process techniques [[8], [9], [10], [11], [12], [13], [14], [15]]. Gravity separation is known to be the oldest and most common employed (pre)concentration process route, which is often conducted on most HM deposits and is usually focused on the rejection of low SG mineral. Nonetheless, the near densities involved in these HMs have posed as major shortcomings during the mineral upgrade, concentration and processing through gravity separations [8,[15], [16], [17], [18], [19], [20], [21]]. Recently, magnetic and electrostatic concentrations seemed very promising and have been tagged very essential physical processing and beneficiation processes employed in exploiting HMs, such as: Zr, Ti, Nb, Ta and most REE bearing HMs from their various mineral deposits [18,22]. Hence, these physical beneficiation and processing techniques which is preferably conducted prior, and flotation processes follows subsequently; have been reported by various researchers to technically upgrade low grade mineral ores or deposits to high grade mineral concentrates as well as improving value metal concentration and recovery from associated mineral impurities in the HM ore deposit. Although several researches have been conducted on the concentration and/or upgrade of HMs, the complexity of certain mineral processing routes as a result of the complex mineralogy of the HM deposits, and the accompanied environmental unfriendliness is still a major constraint in the beneficiation and recovery of HMs. In addition, investigations conducted reveal that the open literature does not completely incorporate the process route, parameters, operating principles as well as the various applications, and as such, comprehensive information in the study area is somewhat scanty, hence, further research investigations. Thus, for successful, feasible, efficient, less complex, cost effective and eco-friendly hydrometallurgical downstream extractions on HM concentrates, the development of proper, optimal mineral processing and physicochemical beneficiation process routes is essentially required. On this premise, this current paper is aimed at reviewing the magnetic and electrostatic mineral processing and beneficiation process methods of HMs for process enhancement and future trend.

## 2 Some HM and their products

### 2.1 Zirconium mineral

The economic concentrations of most HMs, such as: Zr and Ti based or associated minerals can be found in certain primary magmatic mineral deposits as well as being naturally concentrated in sedimentary mineral deposits [1]. Zircon is utilized mostly by the ceramics industries, by the foundry industries as foundry and refractory sand, in the production of television screens as well as a zirconia source in the chemical industries. The Murray Basin of West Australia, Tamil Nadu of India and the sub-saharan of Africa are the world's highest source of these HM sand resources [7]. Zr (Z = 40) is a transition metallic element located between Ti (Z = 22) and Hf (Z = 72) transition metallic elements in group 4B of the periodic table. Similar to niobium (Nb, Z = 41) and tantalum (Ta, Z = 73) transition elements/metals in group 5b of the periodic table, Zr metal usually occur together with Hf in the mineral deposits due to the similarities in physical and chemical properties of both metals. Zr is a shiny, hard and ductile metal which appear very similar to stainless steel. The cold working of Zr with intermediate annealing processes may produce foils, sheets, tubings and bar wires. More so, Zr can also be hot worked on in order to form various rods, slabs, and rounds from an arc melted ingot [23].

It has been established that high purity Zr metal (free of Hf) has a low neutron absorption cross section of approximately 0.18 barns. However, when the pure Zr metal is alloyed with Sn, and with some trace or minute quantities of Fe and Cr, the metal attains certain improved properties such as good formability, high mechanical strength, ductility, and high corrosion resistant in water of elevated temperatures [24]. Zircon mineral is a common accessory mineral in metamorphic and igneous rocks. It is the most important commercial source of Zr and Hf metals, as well as zirconia. In general, it currently cannot be extracted economically, as a result of its low concentration. However, baddeleyite, otherwise referred to as zirkite or zirconium dioxide (ZrO_2_) [1,25] is also regarded an essential source of Zr, containing reasonable Zr amounts. Zr has been reported to be obtained in somewhat small amounts from baddeleyite, which is known to be an accessory mineral in most mafic as well as other silica undersaturated plutonic rocks and dykes [1,26]. For instance, there were reports of the mining of caldesite mineral in 2006 by Cia Brasileira de Aluminio in Brazil [1,27]. This is reported to be a mixture of zircon and baddeleyite minerals [1,28]. However, it was essentially considered as a baddeleyite mineral by Klemic [29].

The industrial and commercial application and consumption of zircon and/or Zr is vast. Generally, the metal Zr is well known for its importance in modern engineering, science and technology [30]. This is a result of its specific unique properties. Zr's special properties such as: high melting temperature point, good hardness, as well as its low thermal coefficient expansivity when heated, makes zircon a very important abrasive material and also a suited foundry or refractory sand [7]. Its resistance to chemical attack and elevated temperatures however makes it a more suitable and efficient refractory material for steel furnaces/ladles and its usage in foundry sands. More so, the utilization of zircon and/or Zr associated HMs in zirconia production as well as the production of Zr metal and Zr based chemicals or compounds. Zr compounds however exhibit different characteristics/properties that make them suitable for several chemical and industrial applications [30]. Primarily, zircon is utilized for its opaque properties. Its resistance to water, chemical, abrasion and heat makes it a very suitable material in certain applications, such as: chemicals, nuclear reactors, foundry, refractories, as well as ceramics [1]. The use of zircon by industries in the production of ceramics such as: porcelain, tiles, sanitary and table wares; is usually consumed or utilized in the form of fine-milled zircon sand mineral. However, zircon mineral with particle size of −45 μm or −75 μm is commercially employed often in frits. Howbeit, zircon mineral is regarded as an effective opacifier as a result of its high refractive index. More so, fine-milled crystals of zircon have the capacity to scatter all the wavelengths of visible light, hence, the reason for the white appearance of ceramics. Further, the high hardness (7.5 Moh scale) property of zircon is also an added benefit that makes it scratch and mechanical damage resistant [30].

Zr has been reported to also find a high utilization in the production of cladding materials for uranium oxide fuel in water cooled reactors by the nuclear power industries as well as a structural material for pressure tube/calandria tube components [24]. Tyler and Minnit [7] reported that over 95 % of the world's recovery of zircon is utilized in the manufacturing of numerous products/compounds of Zr, while the production of Zr metal takes the remaining 5 %. Most of the zircon produced in the world today is applied in the production of ceramics and in ceramics related applications [7]. It was reported by Pownceby et al. [30] that the highest end use of zircon is in the ceramics production by the ceramics industries where it is utilized as glaze opacifiers, opaque frits (a ceramic glass type that is added to glazes for abrasion, water and chemical resistance) and as whiteners for porcelain tiles [30]. This is however as a result of its capability of reflecting and scattering light. The surface layer of most bath-wares, crockery and tiles get their discolouration resistance, durability as well as their glazed finish from zircon melted into the material's surface. Zircon's insensitivity and chemical stability to reducing gases during furnace temperature fluctuations shows that the ceramic engine enhancement is also a developing zircon market [7].

**Fig. 1:**
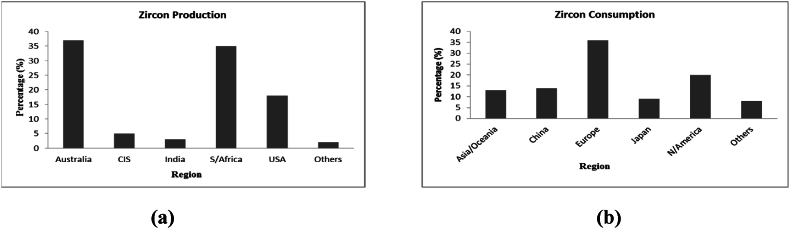
Rate of zircon (a) production and (b) consumption, by regions of the world.

More so, zirconia tends to find various applications in the chemical producing industries. The production of adhesives, antiperspirants, catalyst, dyes, gelatin hardening as well as the manufacturing of aqueous polymers are all applications of zirconia. The chemical compound, potassium hexafluorozirconate (ZrK_2_F_6_ or F_6_K_2_Zr) functions as flame retardants in the textile industries. Zr carbonate (Zr(OH)_2_CO_3_·ZrO_2_) functions as an insolubilizing agent in paper coating companies. Also, Zr oxychloride is utilized in the leather tanning processes [7]. In addition, Zr metal is soft, with high malleability and can be worked upon easily. It possesses a high melting temperature point of about 1670 to 1855 °C as well as high density of 6.49–6.52 g/cm^3^ at room temperature (20–25 °C) and hence, its modern application as superconductors. Zr is an essential tool in the production of television screens due to its capacity to absorb X-rays. Also, the use of Zr in the nuclear industries as control rods can be attributed to Zr's low cross-sectional absorption property. Some other technological utilization of zirconium oxide can be found in the production of oxygen sensors, audio equipment transducers and fuel cells. However, the zircon suitability for any specific application is dependent on the type, quality or specification of the zircon mineral. There are a lot of commercially available, milled products of zircon varying in quality or purity as well as grain size. This zircon quality is classified typically with regards to the content of zirconia as well as the entire mineral composition. For instance, the Fe content of <0.07 % which is referred to as premium grade zircon is often required in the applications of ceramic opacifiers; although the producers of refractory materials have little or no concerns for the Fe content or levels (up to 0.3 %). However, the Fe and alumina content in the minerals are regarded as impurities which negatively affect the opacity characteristics [30,31]. Fig. 1a and 1b respectively summarizes major zircon production and consumption by regions of the world.

### 2.2 Titanium minerals

With almost similar properties or characteristics with zirconium (Zr), titanium (Ti) has a low coefficient of thermal expansivity and combined with a high melting temperature point of 1670 °C and a density between 4.2 and 5.0 g/cm^3^ at room temperature (20–25 °C). Thus, this as a result has made Ti metal and Ti compounds or alloys find several essential applications in the defense and aerospace industries. Ti metal is a light metal (about 45 % lighter than steel), and however two times stronger than aluminium (Al). It can also be machined with similar equipment as that of stainless steel. Under certain atmospheric conditions Ti metal possess corrosion resistant qualities, as it is not easily affected by strong alkali media, sulphides, chlorides and nitric acid media. In addition, Ti metal's good cryogenic characteristics allow its utilization in the production of tanks used for shipping helium, hydrogen or liquid nitrogen [7]. Therefore, the possession of these properties/characteristics has made the application and consumption of Ti metal increase over time, especially in the heat transfer applications (with mild corrosive sea water as coolant), water desalination, oil refineries, and the chemical processing plants. Ti metal has increasingly been utilized in applications of advanced engineering and technology. The manufacturing of jewelries, frames of spectacles and bicycles, sporting goods and the production of the head of golf clubs, are also major applications of Ti metal. As a matter of fact, about 18 % (4750 tons) of the total demand of Ti metal in the year 1996 by United States of America was however attributed to the production of golf clubs [7]. More essentially, the chemical inertness property of Ti metal implies that it may be totally substituting the materials used in conducting prosthetic surgeries in the world of medicine and prosthesis, such as: spinal cord implants, heart pacemakers, dentistry and in hip replacements.

**Fig. 2:**
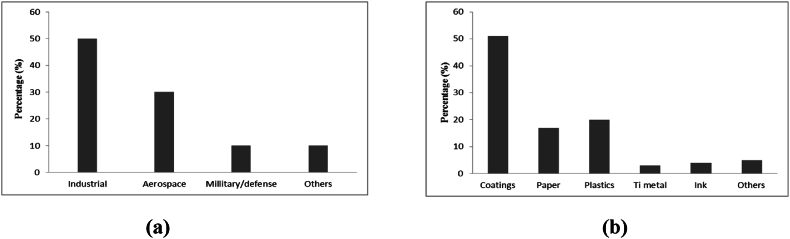
Major consumption of (a) Ti metal, and (b) TiO_2_.

The primary use of titanium feedstocks can be found in certain applications, such as: TiO_2_ pigments and also utilized in metal applications like abrasives, coatings of welding rods, refractory linings and metallurgical fluxes [1]. Over 50 % of the production of TiO_2_ is utilized in the production of pigments in paints, enamels and lacquers. It is utilized in imparting opacity, brightness and whiteness to the production of paper, paints, plastics, etc. The ability of TiO_2_ to absorb UV light tends to slow the paints and plastics degradation, as well as providing its usefulness in sunscreen lotions as inert barrier. It is regarded as an insoluble, non-toxic substance. As a non-toxic, non-fibrogenic and biologically inert compound, TiO_2_ can safely be utilized as filler and whitener in some food items, cosmetics and certain pharmaceuticals. It also possesses good heat stability, hiding power, weather and discoloration resistance, and is easy to disperse in resin systems. Apart from being utilized as white pigments, TiO_2_ can be utilized also in coloured pigments, in colour lightning or tinting to desired measures [1,7,32]. Chemical inertness and high strength are important attributes of Ti utilized by the medical and aerospace industries. In addition, over 95 % of the supply of Ti is for the production of pigments. Perks and Mudd [1] reported that high quality ilmenite, rutile and leucoxene concentrates can directly be utilized as feedstock for the production of TiO_2_ pigment whereas, the lower quality ilmenite needs a prior processing route to synthetic rutile or Ti slag. Producers of Ti sponge may utilize natural rutile, synthetic rutile and slag as well as high quality leucoxene concentrates. However, higher quality leucoxene, natural rutile, reduced ilmenite and slags may be utilized in welding [1]. Certain titanium beneficiation products (especially the products from ilmenite through a chloride media) are usually utilized by the chemical producing companies, and in the production of plastics and paints. Also, the products obtained through the sulphate media are usually applied as a major material in the paper production process by the paper-making industries. More so, the mineral rutile is a very important source of Ti metal and can be utilized as high grade top ups in scenarios of the utilization of increased plants [7]. Finally, the increasing demand and consumption of TiO_2_ as pigments, as well as the recent global disruptions of zircon supply has grown attention in the adequacy of known and unexplored Ti and Zr mineral resources; as well as the ability of the supply to meet the demand in future [1]. Fig. 2a and 2b summarizes rate of demand in major application and/or consumption of Ti metal and TiO_2_, respectively.

## 3 Physical concentration and beneficiation of HMs

**Fig. 3:**
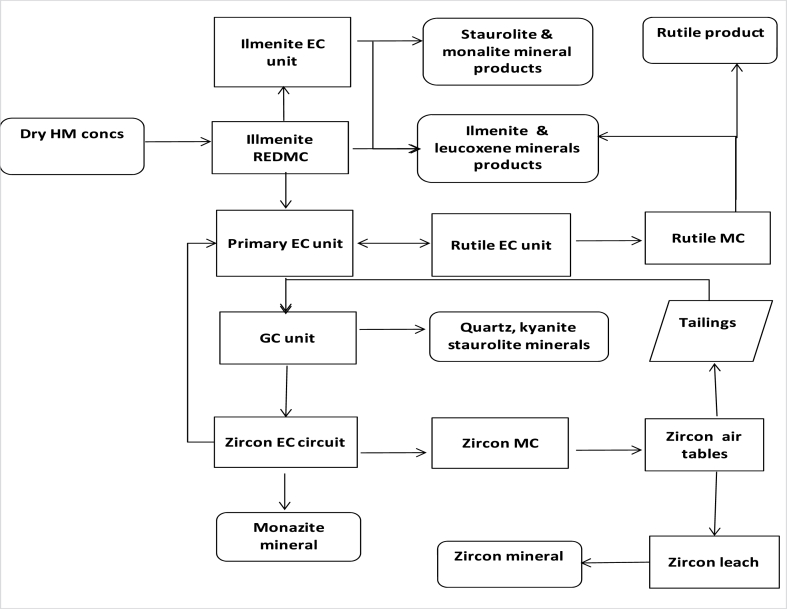
Typical flow diagram of HMs' dry concentration [1,36].

**Fig. 4:**
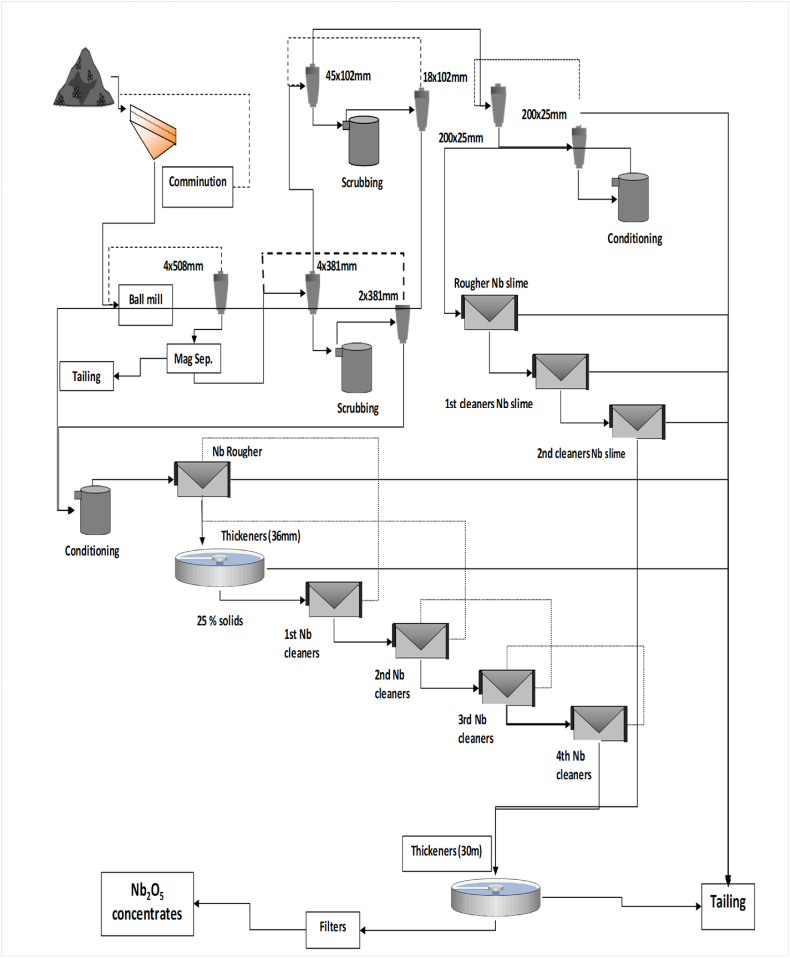
Typical flow diagram of HMs' wet treatment plant [11].

**Fig. 5:**
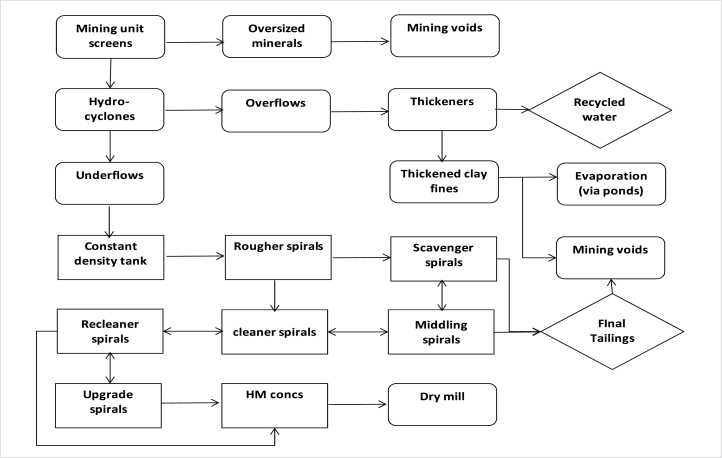
Typical flow diagram of HMs' wet concentration [1,36].

HMs can be got mainly from primary mineral ore deposits or some secondary mineral sources. They often undergo subsequent physical concentration process methods, such as: magnetic, electrostatic, and gravity separations as well as physicochemical concentration techniques such as: froth flotation process. It is therefore noteworthy that the selectivity of these process methods depends greatly on the physical and chemical characteristics and properties of the HMs [[9], [10], [11], [12]]. Physical concentration and beneficiation are usually carried out in order to upgrade the minerals by separating and beneficiating the unwanted impurities or mineral gangue from the VHM. Certain mineral concentrates or products such as: coltan, columbite, pyrochlore, tantalite, rutile, ilmenite, zircon, barite, etc. are primarily realized from such physical processing methods or mineral concentration processes [1,[9], [10], [11], [12]]. Studies conducted by several researchers have shown that the differences in the physical and chemical property existing between the VHMs and other mineral impurities or gangue minerals can be utilized in the separation of value metals from each other and from associated impurities which aids and enhance their recovery and extraction [[9], [10], [11], [12],^33^]. Thus, these mineral impurities contained in the HM ore deposits can be eliminated under certain factors and employing certain conditions such as particle sizes, feed rate as well as specific process parameters, prior to the downstream hydrometallurgical extraction process. This is usually conducted in order to ease up the process complexity and difficulty of the mineral beneficiation and metal extraction process route, thereby possibly reducing the recovery and extraction steps [[9], [10], [11], [12],^33^]. It is therefore very important to conduct ore enrichment and upgrade as well as mineral beneficiation processes on the HM ore deposits for efficient concentration and upgrade to a metallurgical and/or industrially acceptable grade (up to 25–50 % for metals such as: REE, Ti, Hf, Zr, Cu, Pb, Ta, Nb, V, Fe, Zn, Mn, Ba). This will therefore aid the efficiency and effectiveness of the metal recovery and subsequent downstream extraction processes [[9], [10], [11], [12],^23^,^34^,^35^]. Dry or wet physical concentration process techniques can either be employed. Thus, the flow diagrams of dry and wet concentration techniques are displayed in Fig. 3, Fig. 4, Fig. 5.

### 3.1 Magnetic concentration

The high intensity magnetic concentrators attract the ferromagnetic mineral particles and highly paramagnetic mineral particles as concentrates, separating them from the rest of the mineral particles. Meanwhile, the diamagnetic or non-magnetic mineral particles are realized as tailings. In most times, weak paramagnetic mineral particles may also tend to be attracted to high intensity magnets in the magnetic concentrators. This may depend chiefly on the measure of magnetic intensity of the involving magnets and the minerals’ particle size; as it is noteworthy that coarse mineral particles are more susceptible or amenable to magnets than the finer particles. On the other hand, low intensity magnetic concentrators tend to attract and therefore separate the ferromagnetic mineral particles as concentrates, whilst the paramagnetic and diamagnetic mineral particles are realized as tailings. Often times, low intensity magnetic separators tend to be adequately and sufficiently effective on highly or strong paramagnetic minerals with coarse particle sizes and will also realize these mineral particles as concentrates. More so, the finer highly or strong paramagnetic, weak paramagnetic as well as diamagnetic mineral particles are realized as tailings [9,11]. Table 1 enumerates different HMs and their respective magnetic susceptibility and electrostatic responses.

**Table 1:** Certain HMs and their magnetic susceptibility and electrostatic responses [[1], [22], [37], [38], [39], [40], [41], [42], [43], [44], [45], [46], [47]].

HM name	General chemical formula	Response to magnetism (Magnetic susceptibility)	Response to electrical conductivity
Actinolite	Ca_2_(Mg,Fe)_5_(Si_4_O_11_)_2_(OH)_2_	2	b
Almandine	Fe_3_Al_2_(SiO_4_)_3_	2	b
Amphibole	(Fe,Mg,Ca)_x_SiO_3_	2	b
Anatase	TiO_2_	3	a
Andalusite	Al_2_SiO_5_ Polymorphs	3	b
Andradite	3CaO·Fe_2_O_3_·SiO_2_	2	b, a
Anhydrite	CaSO_4_	3	b
Ankerite	Ca(Mg,Fe)(CO_3_)_2_	2	b
Apatite	(F_1_Cl_1_OH)Ca_5_(PO_4_)_3_	3	b
Aragonite	CaCO_3_	3	b
Arsenopyrite	FeAsS	2, 3	a
Augite	Ca(Mg,Fe,Al)[(Si,Al)2O_6_]	2	a, b
Azurite	Cu_3_[CO_3_]_2_(OH)_2_	3	b
Baddeleyite	ZrO_2_	3	b
Barite	BaSO_4_	3	b
Bastnasite	(Ce,La)(CO_3_)F	2	b
Biotite	K(Mg,Fe)_3_[Si_3_AlO_10_](OH,F)_2_	2	b
Bismuth	Bi	3	a
Bornite	Cu,FeS4	3, 2	a
Brannerite	(UO,TiO,UO_2_)TiO_3_	2	a
Brookite	TiO_2_	3	a
Carnotite	K_2_(UO_2_)_2_V_2_O_8_·2H_2_O	3	b, a
Cassiterite	SnO_2_	3	a
Celestite	SrSO_4_	3	b
Cerussite	PbCO_3_	3	b, a
Chalcocite	Cu_2_S	3	a
Chalcopyrite	CuFeS_2_	3, 2	a
Chlorite	(Mg,Al,Fe)_12_[(Si,Al)_8_O_20_](OH)_16_	2	b
Chromite	(Fe,Mg)(Cr,Al)_2_O_4_	2	a
Cinnabar	HgS	3	b
Cobalitite	(Co,Fe)AsS	2	a
Columbite	(Fe,Mn)(Nb,Ta)_2_O_6_	2	a
Corundum	Al_2_O_3_	3	b
Covellite	CuS	3	a
Cryolite	Na_3_AlF_6_	3	b, a
Cuprite	Cu_2_O	3	b
Diopside	CaMg[Si_2_O_6_]	2, 3	b
Epidote	Ca_2_(Al,Fe)_3_Si_3_O_12_(OH)	2	b
Eudialyte	Na_4_(Ca,Ce)_2_(Fe^2+^,Mn^2+^,Y)ZrSi_8_O_22_(OH,Cl)_2_	3	b
Euxenite	(Y,Ca,Ce,U,Th)(Nb,Ta,Ti)_2_O_6_	2	a
Ferberite	FeWO_4_	2, 1	a
Fergusonite	(REE)(Ba,Nb,Ta)O_4_	2	a
Fluorite	(Ca,REE)F_2_	3	b
Franklinite	(Zn,Mn)Fe_2_O_4_	1	a
Gahnite	ZnAl_2_O_4_	3	b
Galena	PbS	3	a
Garnet	Ca,Mg,Fe,Mn silicates	2, 3	b, a
Geothite	FeO(OH)	2	b, a
Grossularite	Ca_3_Al_2_(SiO_4_)_3_	3	b, a
Hematite	Fe_2_O_3_	2	a
Homblende	Ca_2_Na(Mg,Fe^2+^)_4_(Al,Fe^3+^)[(Si,Al)_4_O_11_](OH)_2_	2	b, a
Huebnerite	MnWO_4_	2, 3	a
Hypersthene	Mg,Fe)SiO_3_	2	b
Ilmenite	Fe^2+^TiO_3_	1, 2	a
Ilmenorutile	(Nb_2_O_5_,Ta_2_O_5_)_x_TiO_2_	2	a
Ilvaite	CaFe_2_(FeOH)(SiO_4_)_2_	2	a, b
Kyanite	Al_2_O[SiO_4_]	3	b
Leucoxene	FeTiO_3_	2, 3	a
Loparite	(Ce,Na,Ca,La,Sr)_2_(Ti,Nb)_2_O_6_	2	a
Magnesite	MgCO_3_	3	b
Magnetite	Fe_3_O_4_	1	a
Malachite	Cu_2_CO_3_(OH)_2_	3	b
Manganite	MnO(OH)	2, 3	a
Marcasite	FeS_2_	3	a
Microlite	(Na,Ca)_2_Ta_2_O_6_(O,OH,F)	3	b
Millerite	NiS	2	a
Molybdenite	MoS_2_	3	a
Monazite	(Ce,La,Y,Nd,Th)PO_4_	2	b
Mullite	Al_6_Si_2_O_13_	3	b
Muscovite	KAl_2_[AlSi_3_O_10_][F,OH]_2_	3	b
Niccolite	NiAs	2	a
Olivine	(Mg,Fe)_2_[SiO_4_]	2	b
Orpiment	As_2_S_3_	3	a
Periclase	MgO	3	b
Perovskite	(Ca,REE)TiO_3_	3	b
Pyrite	FeS_2_	3, 2	a
Pyrochlore	(Na,Ca.)_2_(Nb,Ta.)_2_O_6_[O,OH,F]	3	a
Pyrolusite	MnO_2_	3, 2	b
Pyrope	Mg_3_Al_2_(SiO_4_)_3_	3	b, a
Pyroxene	(Ca,Mg,Fe,Al)_2_Si_2_O_6_	2, 3	b, a
Quartz/silica	SiO_2_	3	b
Realgar	AsS	3	a
Rhodochrosite	MnCO_3_	3	b, a
Rhodonite	MnSiO_3_	3	b, a
Rutile	TiO_2_	3	a
Samarskite	(Fe,Ca,U,Y,Ce)_2_(Nb,Ta)_2_O_6_	2, 1	a
Scheelite	CaWO_4_	3	b
Siderite	FeCO_3_	2	b, a
Sillimanite	Al_2_O[SiO_4_]	3	b
Smithsonite	ZnCO_3_	3	b
Spessarite	Mn_3_Al_2_[SiO_4_]_3_	3	b
Sphalerite	ZnS	2, 3	a, b
Sphene	CaTi[SiO_4_](F_3_OH)	3	b, a
Spinel	MgAl_2_O_4_	3, 2	a
Spodumene	LiAl(SiO_3_)_2_	3	b
Stannite	Cu_2_FeSnS_4_	3	a
Staurolite	Fe^2+^Al_4_[Si_4_O_11_]_2_O_2_(OH)_2_	2	b, a
Stibnite (Antimonite)	Sb_2_S_3_	3	a
Struverite	(Ti,Ta,Nb,Fe)_2_O_6_	2	a
Tantalite	(Fe,Mn)(Ta,Nb)_2_O_6_	2	a
Tapiolite	(Fe,Mn)(Nb,Ta,Ti)_2_O_6_	2	a
Tetrahedrite	(Cu,Fe)_12_Sb_4_S_13_	2	a
Thorianite	ThO_2_	3	b
Thorite	ThSiO_4_	3	b
Topaz	Al_2_SiO_4_(F,OH)_2_	3	b
Tourmaline	(Na,Ca)(Mg,Fe^2+^,Fe^3+^,Al,Li)_3_Al_6_BO_3_)_3_Si_6_O_18_(OH)_4_	2, 3	b, a
Uraninite	UO_2_	2	b
Wolframite	(Fe,Mn)WO_4_	2	a
Wulfenite	PbMoO_4_	3	a
Xenotime	YPO_4_	2	b
Zincite	ZnO	3	b, a
Zircon	(Zr,REE,Hf)SiO_4_	3	b

Key: Ferromagnetic = 1; Paramagnetic = 2; Diamagnetic = 3; Conductive = a; Non-conductive = b.

### 3.2 Magnetic separators

**Fig. 6:**
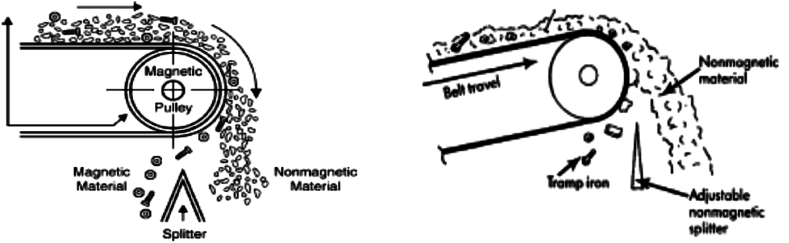
Magnetic pulley [40,49].

Magnetic separators have been reportedly useful in separating somewhat coarser mineral particles. Over the past decades, there have been unusual development of magnetic separators and hence, the advancement of various classes of magnetic separators as well as different equipment designs that concentrate and beneficiate HMs [[9], [48], [49]]. The most practical and logical class is either the dry or wet magnetic separators. However, the mechanism of these magnetic physical separators is reported elsewhere [9]. These physical separators can either operate with low or high magnetic field intensities [9,48,49]. There have been several reports that the gradient of magnetic field is not considered in this situation; and hence, one can say that the high intensity magnetic separators (HIMS) may generate high magnetic field gradient and usually referred to as high gradient magnetic equipment; whilst low intensity magnetic separators (LIMS) may be referred to as low gradient magnetic equipment, and generates low magnetic field gradient [[9], [49], [50], [51], [52], [53], [54], [55], [56], [57], [58], [59], [60], [61]]. Nonetheless, this rule may have some exceptions. In that regard, the choice and classification of magnetic separators may be dependent on several factors or considerations, most essentially the particle size, distribution of the feed mineral particles, as well as their magnetic characteristics and the throughput of the required equipment [[9], [49], [62]]. Fig. 6 displays typical schematic diagrams of a (low-intensity) magnetic pulley for the separation of highly magnetic mineral particles from the non-magnetic particles.

**Fig. 7:**
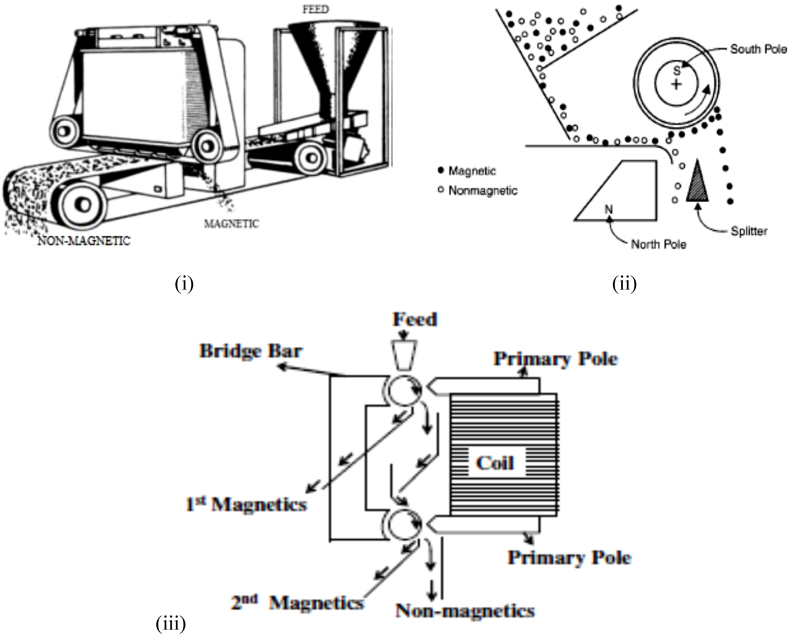
Electro-magnetic concentrators (i) cross belt (ii) lift roller (iii) induced roller.

**Fig. 8:**
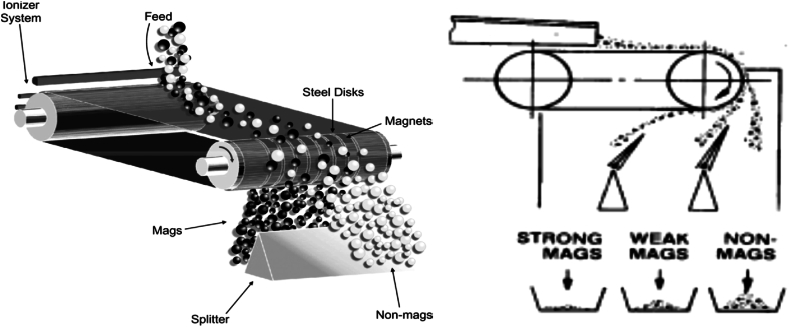
RER magnetic concentrator [38,40,49,62,64].

**Fig. 9:**
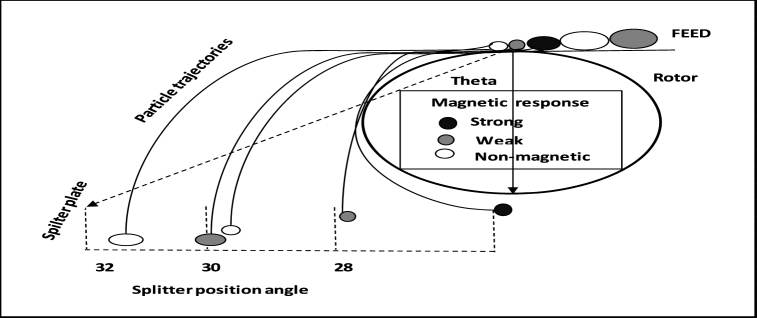
Trajectories of HM particle size fractions and their magnetic susceptibility [38,62,65].

Wet MS procedure was however reported to require further processing by Nzeh et al. [9] and Zong et al. [62]. But in contrast, dry MS was reported to possess more merits when compared to the wet MS. For instance, in comparison to dry MS, wet MS involves water consumption, the reuse of recycled water, and the mineral tailings’ pond management, etc. Other than dry and wet magnetic separators, other classes of magnetic separators may exist, and may be grouped based on the magnetic intensity of the separator or the strength of their magnetic field. This can also be further divided into electro-magnetic separators, and may include: (i) cross belt magnetic separators (CBMS) (ii) lift roller magnetic separators (LRMS) (iii) induced roller magnetic separators (IRMS) (Fig. 7) and permanent magnetic separators, which may include: (i) rare earth roll magnetic separators (RERMS) (Fig. 8) and (ii) rare earth drum magnetic separators (REDMS). These have been discussed in details by several researchers [9,11,38,40,62,63]. Fig. 8, Fig. 9 respectively depicts the RER magnetic concentrator procedure of concentrating mineral particles, and the particle trajectories of the different size fractions of mineral particles and their magnetic susceptibility.

**Fig. 10:**
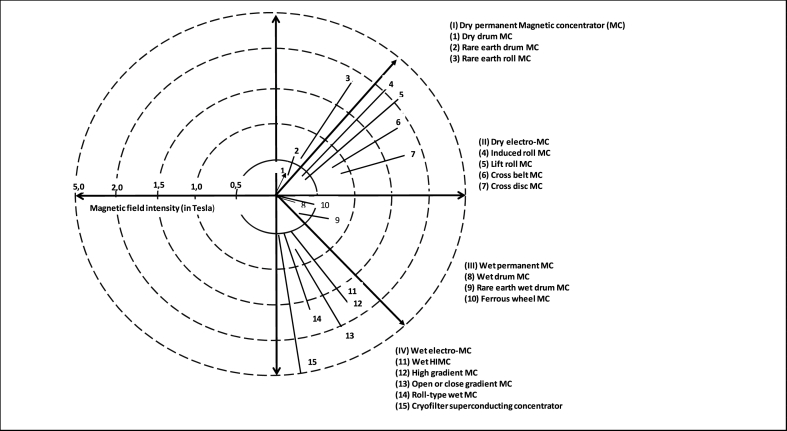
Working range of different magnetic concentrators used by mineral industries [38,66].

Effect of particle size of the feed mineral on the efficiency of a particle size-based RER magnetic separator is schematically displayed in Fig. 8, Fig. 9. The trajectories of nonmagnetic mineral particles are simply achieved by centrifugal force (the force obtained by centrifuge). When the nonmagnetic particles of a HM, such as zircon mineral, drops in an unhindered movement and substantially in the roller according to the particle size distribution (PSD) of the HM, the drop of the particles nearer to the roller tend to reduce their size fractions (as in Fig. 9). However, the larger HM particles may tend to move far away from the roller smaller centerline, and most times lower than surface velocity of smaller HM particles concentrated. Howbeit, when a magnetic roller is used, the magnetic HM particles that have somewhat higher magnetic fields may tend to likely stick on the surface of the magnetic roller until they are released from the field of magnetic force. In addition, magnetic HM particles that have somewhat weak magnetic field will be defected by magnetic field, thereby causing them to likely deviate from the normal path; hence, occurrence of overlapping in coarser weak magnetic HM particles and finer nonmagnetic HM particles [9,62]. Fig. 10 displays the different working range of various magnetic separators that are being employed by mineral processing and extraction industries.

### 3.3 Conventional or low intensity magnetic concentrators (LIMS)

LIMS are conventional magnetic separators mainly employed in eliminating ferromagnetic HM (which basically includes Fe) or some highly paramagnetic HMs. This is usually conducted majorly to scalp the very magnetic HM particles in order to improve performance of permanent magnets and electro-magnetic separators often utilized in separating weak magnetic materials and/or to protect downstream process operations, such as conveyor belts. These separators may make use of flux densities (of approximately 2000 Gauss) and has the capability of treating wet slurry and dry solids [9,40,58].

#### 3.3.1 Dry and wet LIMS

**Fig. 11:**
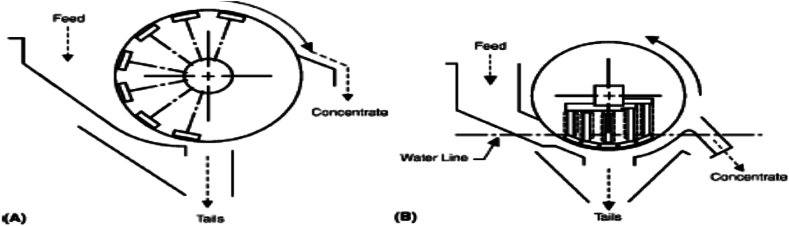
(a) Counter-rotation (b) Concurrent-rotation magnetic drum separators [9,40].

Application of dry LIMS is often employed in the separation of highly magnetic VHMs and/or in eliminating highly magnetic gangue or mineral impurities and tramp iron. More so, dry LIMS is usually employed in separating coarser HM sands that are composed of highly magnetic HM particles. This process is basically known as cobbing [9,49]. The magnetic drum separators are often used in dry LIMS, for physically separating highly magnetic VHMs whilst the plate and grate magnets, suspended magnets and magnetic pulleys are used mainly in the removal of gangue or mineral impurities and tramp iron [49]. However, below 500 particle size, the dry LIMS is often times replaced with wet LIMS. In addition, drum separators are known to be the most commonly used wet LIMS, with counter-rotating and concurrent-rotating magnetic drum separators (Fig. 11) as the major common types. With introduction of the permanent ferrite magnets, permanent magnetic system almost replaced electro-magnetic drum system. Therefore, the separators are basically used in the beneficiation and recovery of HM media, such as: zircon HM sand, ferrosilicon, and in magnetic media for separating highly magnetic HM ores, such as magnetite HMs and ferromagnetic HM sands. Moreover, existence of rare earth permanent magnets (REPM) and its cost efficiency and affordability tend to increase the application of magnetic drum separators on moderate or weak magnetic HM particles [9,40,49]. Figs. 11a and 11b shows diagrams of counter-rotation (non-submerged magnetic field) and concurrent-rotation (submerged magnetic field) magnetic drum separators, respectively.

**Fig. 12:**
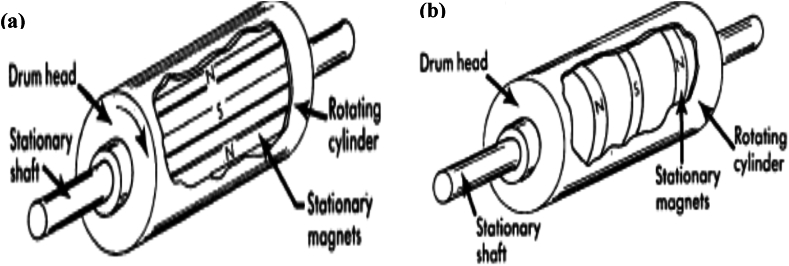
(a) Axial poles alternating along the circumference (b) Radial poles alternating across the drum breadth [49].

Technically, there are two design types of permanent magnetic drum separators which may include the radial and axial configurations. Radial arrangement or configuration may involve polarity of permanent magnets alternating across the breadth of the drum; and its application importantly involves the recovery of highly magnetic HM particles. On the other hand, axial arrangement or configuration may involve the alternation of poles along the circumference. Its application is usually preferred when magnetic product quality is of somewhat actual significance. In addition, tumbling motion of HM particles over certain rows of magnets coupled with alternate polarity may tend to assist or aid the release of nonmagnetic HM particles entrained and thus, the concentrate grade is enhanced [40,49]. Fig. 12a and 12b shows diagrams representing the axial and radial poles, respectively.

### 3.4 High intensity magnetic separators (HIMS)

Reports have established the knowledge that paramagnetic HM particles are somewhat difficult, tedious, and almost not feasible to beneficiate using LIMS, hence, require the use of HIMS process. These separators require a higher magnetic flux densities up to 20,000 Gauss (approximately 2 T) so as to separate the weakly magnetic (paramagnetic) HM particles from nonmagnetic HMs [9,40,58].

#### 3.4.1 Dry and wet HIMS

It is reported that HIMS are used in various applications by different mineral processing and extractive metallurgical industries [38]. Thus, sustaining and emerging research that is based on the performance evaluation of wet and/or dry HIMS employed in the beneficiation and concentration of various HMs is inevitable. Nevertheless, there have been continuous attempts by different researchers and technologists over the years, in order to completely understand the physical beneficiation and separation behavior of various HM particles in the wet and/or dry HIMS process techniques, thereby concentrating the various HM types with different magnetic susceptibility levels. Thus, the use of HIMS in numerous application has been greatly influenced by the inability of LIMS to beneficiate paramagnetic HMs over the decades. On this premise, the research by Tripathy et al. [38], was concluded on the note that dry HIMS are yet to find somewhat wide applications in the beneficiation and concentration of some (paramagnetic) HMs, despite the intense work and efforts or investigations and studies carried out by a lot of researchers. Over the years, the dry HIMS application has undergone exceptional technological and engineering advancements or developments. Consequently, there have been the emergence of various approaches to novel applications and designs of HIMS. Thus, over the decades, several effective and efficient types of magnetic separators have evolved with several varying designs and of wide range of industrial importance. The open literature has also established that magnetic separation can be employed as an integral step of primary and/or secondary system unit in achieving pre-concentration and/or primary concentrations and beneficiations of HM particles. Therefore, dry HIMS applications have been accepted as ideal magnetic separation process technique in selectively beneficiating or physically separating and/or recovering paramagnetic HM particles from different HM deposits or sources [38,[67], [68], [69]]. In addition, most dry HIMS may possess higher magnetic field intensity imparted through the induced magnetic fields or by a permanent magnet for the separation of HM particles with respect to their magnetic susceptibility level [9,38]. Table 2 depicts major HIMS, their most essential characteristics, varying parameters or variables and most common applications on HMs.

**Table 2:** Major HIMS, influencing variables, characteristics, and mineral applications [38].

Separators	Max. magnetic strength or intensity	Variables	Key characteristics	Mineral applications
Permanent roll/RERMS	1.6 T	Feed rate, belt thickness, roll disk thickness, roll diameter, roll speed, splitter position, roll magnetic intensity, magnetic susceptibility & PSD.	Can be employed at various stages of concentration. Employed in higher capacities & with better efficiencies. Low belt life & requires frequent replacements.	Ilmenite, feldspar, (sub)bituminous coal, lignitic fly ash, bauxite, quartz/silica sand, lignite, perlite, colemanite, nepheline syenite, diamond, coal, semi coking coal, trona, wollstonite, Mn mierals, etc.
CBMS	2 T	Feed rate, applied current, main belt speed, pole air gap, coil current, cross belt speed & thickness, magnetic susceptibility & PSD.	The unit capacity is somewhat very low. It is utilized mostly as a cleaner process step.	Columbite, coltan, pyrochlore, tantalite, microlite, zircon, albite, cobalt, BOF & LD slags, Fe & Mn minerals, etc.
IRMS	2 T	Feed rate, rotor speed, rotor diameter, gap between rotor and pole, applied current, splitter position, magnetic susceptibility & PSD.	The magnetic intensity/strength can be varied. Can be utilized as pre-concentrator, cleaner or scavenger. Single unit process may be utilized in multi stage feed PSD of 3 mm (coarser) and 75 μm (finer) mineral particles. Very low unit capacity.	Hematite, ilmenite, dolomite, garnet, magnesite, nepheline syenite, plant tailings, Cr, Mn, Sn & W minerals, etc.
LRMS	2 T	Feed rate, rotor speed, rotor diameter, gap between rotor and pole, applied current, splitter position, magnetic susceptibility & PSD.	Very low unit capacity. Utilized mostly as a cleaner. Magnetic intensity/strength can be varied. Feed PSD of 3 mm (coarser) and 75 μm (finer) mineral particles.	Garnet & ilmenite, etc.

Apart from the HIMS techniques depicted in Fig. 10, some other magnetic separators that may fall under the category of dry HIMS have also been studied over time by several researchers. They may include: permanent roll, open gradient, superconducting high gradient, vibrating high gradient filter and isodynamic magnetic separators [9,38,39,49,67,68,[70], [71], [72], [73], [74], [75], [76], [77], [78], [79], [80], [81]]. However, the isodynamic separator or tester is mostly used primarily to achieve quantitative analysis of paramagnetic HMs. More so, it has been widely used in characterizing HMs, measurement of magnetic susceptibility and in the stimulation of grade and recovery curve. On the other hand, high gradient high intensity magnetic separators (HG-HIMS) and open gradient high intensity magnetic separators (OG-HIMS) have not successfully been commercialized [9,38]. This was however due to their inefficient magnetic field strength or intensity and also attributed to the lack of uniform distribution of magnetic induction over the magnetic drum surface. Nonetheless, HG-HIMS has been utilized in laboratory scales, majorly in the separation or elimination of pyrite HMs from coal fines; it was therefore found inefficient regardless [9,38,44,76,77,[82], [83], [84], [85], [86]]. However, research is still scanty in these areas (OG-HIMS and HG-HIMS) for effective and cost efficient magnetic separation.

The dry HIMS is importantly used in the separation of somewhat coarser HM particles, although the wet process is required subsequently as an adjunct process. Both wet and dry HIMS may have certain demerits and merits over each other. Dry HIMS is more advantageous over wet HIMS in the sense that it does not require water consumption, retrieval of water for re-use, management of tailings ponds, and also there is less energy consumed so much so that pre-concentration is conducted on feed HM particles of coarser size fractions or PSD prior to subsequent fine grinding for larger HM liberation with lesser volume. In addition, it is noteworthy that dry HIMS has certain limitations or demerits which are regarded as merits for wet HIMS. These may include: control of dust particles so as to avoid air or atmosphere pollution, inefficiency in concentrating, treating and separating fine and/or ultrafine HM particle size fractions and the removal of slime coating on coarser HM products. Dry HIMS is thus, a more efficient and effective physical separation and beneficiation process of HM particles with size fractions or PSD greater than −75 + 100 μm. This is not the case with wet HIMS, as the wet HIMS is regarded a more efficient process technique for the physical separation and beneficiation of HM particles with size fractions or PSD below −75 + 100 μm on most paramagnetic HM particles [9,38].

However, there have been reports that the manufacturers of dry magnetic separators were on top of the production of magnetic equipment that beneficiates and concentrates placer beach sand particles or HM sand particles of size fraction of 45 μm [9]. Therefore, in the course of addressing the dust challenges involved in dry magnetic separators, different process methods have been developed and employed by several researchers over the years. These process methods may include mineral de-dusting procedure prior to subsequent separation and addition of purge air between feed and separation/concentration sections of the magnetic separators [9]. Recently, several measures have been taken in order to address the challenges and curtail the demerits of HIMS. Such measures were in the HIMS design which may include design of the hopper, the door, as well as the frame systems of HIMS. These were done in order to enable the containment of the dust particles in the equipment during the HM physical separation process. These units were therefore internally equipped with the design of dust extraction (per stage), tunable as well as purge air in cassettes so as to reduce dust problems to a minimum. More so, in addressing the environmental challenges and eco problems caused by dust pollution, a deeper, dust bag filter equipped with fluidized dust catchers may be implemented as an integral part of the magnetic separation system. However, the economics of this procedure may not be favorable [9,38,87]. It was therefore concluded by Dobbins et al. [87] in their reports that the combinations of these process steps are regarded as improvements and enhancements to better, cleaner and safer environment, as well as the improved part life and sustainability of the performance of such magnetic separators. Table 3 therefore summarizes applications of various magnetic separators on certain HMs as investigated by several researchers.

**Table 3:** Different applications of various magnetic separators on HMs

Separator	HMs	Investigated variables	Details	Citation
IRMS	Brazilian zircon mineral concentrates	–	Elimination of magnetic ilmenites from zircon	Sampaio et al. [88]
	Indonesian high grade zircon-rich bearing HM concentrates	–	Part of a flow diagram as a pre-concentration process of zircon & ilmenites	Aral et al. [89]
	Wolframite-scheelite-cassiterite mineral in Kyrgyzstan	–	Pre-concentrating Sn & W	Sreenivas et al. [90]
	Garnet fines	Feed rate, PSD, magnetic intensity, splitter position & rotor speed	Understanding the effects of PSD	Tripathy & Suresh [91]
	Indian Hematites	Feed rate, PSD, magnetic intensity, splitter position & rotor speed	Separating hematite fines low grade fines	Tripathy et al. [65]
	Egyptian Magnesite-dolomite	Rotor speed & magnetic intensity	Separating Fe associated magnesite from dolomite	Yehila & Al-Wakeel [92]
	Indian ferruginous chromites	Rotor speed & magnetic intensity	Separating ferruginous mineral impurities/gangues	Tripathy et al. [93]
	Turkish chromite tailings	Feed rate, rotor speed & magnetic intensity	Cleaning and recovering value minerals	Aslan & Kaya [94]
	Indian Mn ores	Rotor speed, PSD & magnetic intensity	Enhancing Mn:Fe ratios by desliming & beneficiation	Tripathy et al. [95]
	Turkish nepheline syenite ores	Feed rate, PSD, magnetic intensity, splitter position & rotor speed	Rejecting Fe associated mineral impuries/gangues	Ibrahim et al. [96]
	Indian Hematites	Feed rate, rotor speed & magnetic intensity	Separating hematite fines low grade fines	Tripathy et al. [63]
	Sudan Fe ores	Rotor speed, PSD & magnetic intensity	Part of a flow diagram as rougher & cleaning process	Seifelnassr et al. [97,98]
	Indian chromite tailings	Rotor speed & magnetic intensity	Part of a flow diagram in order to enhance Cr:Fe ratio	Tripathy et al. [99]
	Indian ferruginous Mn ores	PSD, magnetic intensity, splitter position & rotor speed	Rejecting Fe associated mineral impurities/gangues	Singh [100]
	Indian ferruginous chromites	Rotor speed & magnetic intensity	Cleaning and recovering value minerals	Tripathy et al. [101]
	Indian low grade ferruginous Mn ores	Rotor speed & magnetic intensity	Part of a flow diagram as a (2-stage) separation process	Singh et al. [102]
CBMS	South Korean fine dredged aggregate waste	–	Concentrating and recovery of the value HMs (zircon, ilmenite, quartz, muscovite, monazite, magnetite, etc.)	Moscoso-Pinto & Hyung-Seok [103]
	Nigerian chromites	–	Enhancing Cr: Fe ratios	Abubakre et al. [104]
	Ti associated minerals in Sri Lanka	Magnetic intensity	Disk-type separation of Ti associated minerals from beach sand minerals	Premaratne and Rowson [105]
	Nigerian columbites	–	Pre-concentrating process	Ayeni et al. [106]
	Slags from basic O_2_ furnace steel making	Magnetic intensity	Separating Fe associated phases	Wang et al. [70]
	Nigerian columbites	PSD, single & double stages	Part of a flow diagram as a 3 disk-type separation of columbites from impurities	Alabi et al. [58]
	Egyptian Albite ores	Feed rate & magnetic intensity	Separating Fe and Ti compounds as impurities	El-Rehiem & El-Rahman [107]
	Indian low grade siliceous Mn ores	Magnetic intensity & PSD	Separating Mn associated mineral particles	Mishra et al. [108]
	Indian cobalt associated Mn ores	Magnetic intensity & PSD	Separating Co associated Mn mineral particles	Mishra et al. [109]
	Egyptian low grade Fe ores	PSD	Upgrading Fe composition of low grade mineral ore	Al-Wakeel & El-Rahman [110]
	French Linz-Donawitz slags	Magnetic intensity	Separating Fe associated phases	Menad et al. [111]
RERMS	Silica mineral sand	Splitter angle position, belt speed & PSD	Hematite removal	Ibrahim et al. [112]
	Turkish colemanites	Feed rate, splitter angle position, roll speed & PSD	Separating colemanites from impurities/gangues	Alp [113]
	Indian hematites	Feed rate, magnetic roll, Splitter angle position & roll speed	Separating hematite fines from low grade fines	Tripathy et al. [114]
	Turkish semi coked lignite	Feed rate, front & back splitter angle position & roll speed	Reduction of ash & sulphur composition	Atesok et al. [115]
	Semi coked lignite of low grade	Feed rate, splitter angle position, roll speed & PSD	Rejection of sulphur	Yildinm et al. [116]
	Ferruginous chromite mineral ores	Feed rate & roll speed	Separating ferruginous mineral impuritiues/gangue	Tripathy et al. [93]
	Indian bauxite mineral ore	PSD & roll speed	Rutile, hematite, & goethite mineral separation	Bhagat et al. [117]
	Indian low grade Mn siliceous mineral ores	PSD	Upgrade of Mn composition	Dash et al. [118]
	Ferruginous chromite mineral fines	Feed rate & roll speed	Enhancing Cr:Fe ratios by Fe bearing mineral removal	Tripathy et al. [66]
	Indian Teri sand, Ilmenite minerals	–	Ilmenite magnetic mineral concentration	Babu et al. [119]
	Turkish low grade coals	–	De-sulphurization & de-ashing procedure	Celik [120]
	Turkish Mn mineral ores	PSD	Separation of Mn associated minerals	Atesok et al. [121]
	Low grade Fe siliceous ore fines	PSD	Upgrade of Fe composition	Dwari et al. [122,123]
	Indian charged chrome slags	Roll speed	Separation of chrome metal from slags	Shen & Forssberg [124]
	Turkish lignite fly ash	Roll speed & PSD	Pre-concentration & separation of Fe associated compounds/impurities	Ozdemir & Celik [125]
	(Sub)bituminous coal	–	Separation of shale	Oder [126]
	Indian charged chrome slags	Roll speed	Separation of chrome metal from slags	Das et al. [127]
	British (UK) coals	–	Pyrite separation from coal	Saeid et al. [128]
	Trona in USA	Splitter angle position & PSD	Trona pre-concentration & elimination of illite & dolomitic shale	Ozdemir et al. [129]
	Lignite in USA	–	Separation of macerals	Order et al. [130]
	Turkish lignite	Feed rate, splitter angle position, roll speed & PSD	Rejection of sulphur	Koca et al. [131]
	Egyptian nepheline syenites	Feed rate, PSD & rotor/belt speed	Removal of Fe associated mineral impurities/gangues	Ibrahim et al. [96]
	Greek bauxite mineral ore	–	Separation of calcite from bauxite	Stamboliadis & Kailis [132]
	Turkish Mn mineral ore	PSD	Separation of braunite	Grieco et al. [133]
	Turkish feldspar	Magnet:steel ratio & PSD	Removal of Fe associated mineral impurities/gangues	Gülsoy & Orhan [134]
	Perlite in Israel	–	Removal of Fe associated mineral impurities/gangues	Herskovitch & Lin [135]
	Turkish feldspar	PSD	Separation of coloured magnetic particles	Gülsoy et al. [136]

## 4 Electrostatic concentration

Electrostatic concentration is a physical separation technique usually employed on HMs with the aid of electrostatic fields, where electrical forces tend to act on the charged solid mineral particles in an electric field. This mineral concentration technique comprises of various methods that could be applied on different scales and on different minerals for various purposes [137]. Electrostatic concentration of primary, secondary and HM sand or alluvial mineral deposits have been established in the open literature and also industrially (especially the industrial utilization of high-tension roll separators) [11,[138], [139], [140], [141], [142], [143], [144], [145]]. Electrostatic concentration is however mostly a dry concentration process of HMs and unfortunately employs a pre-heating treatment process (of temperatures between 100 °C to 150 °C) in order to remove humidity and also prepare the feed temperature [138]. Several researchers have investigated and reported the adoption of electrostatic concentration on HM deposits, mineral sands or alluvial deposits during physical processing/beneficiation of such minerals, describing the general principles as well as the application for physical separation or beneficiation purposes [11,[137], [138], [139], [140], [141], [142], [143], [144], [145], [146], [147], [148], [149], [150], [151], [152]]. However, in addition to the required heat treatment process which involves a certain level of energy consumed during the process, almost all HM sands and RE mineral deposits requires a prior comminution process in order to grind the mineral deposit to particles and prepare them for concentration. This is associated with energy costs and are also somewhat cost intensive for such process to be utilized in a commercial or industrial level [15].

Also, it has been established by researchers that wet physical processing techniques of minerals such as gravity concentration and also froth flotation methods have dominated the industries of the physicochemical mineral processing of HMs. However, electrostatic concentration technique has been reported by several researchers to have a somewhat more wide application on HMs and mineral sands such as: rutile, ilmenite, leucoxene, hematite, coal (electrical conductors), zircon, quartz, monazite, kyanite, staurolite, sillimanite, garnet (non-conductors), than the wet mineral processing techniques [36,137,[153], [154], [155], [156]]; and its application is considered more advantageous than the wet mineral processing techniques especially in geographical areas with limited water resources or with the short supply of water [154,157]. According to the numerous reports from these researchers, such minerals must show or respond to a certain degree or level of electrical conductivity and non-conductivity. Hence, this mineral processing or beneficiation procedure has been reported to involve the utilization of electrostatic concentrators. These concentrators exploit the electric force fields in the mineral particles and utilize the electrical conducting property difference of the various mineral particles during separation. Thus, electrical conducting mineral particles (conductors) such as ilmenite and rutile are being separated from the non-electrical conducting minerals (non-conductors) such as zircon, quartz and monazite by employing either low or high tension electrical concentrators. This is however dependent on the degree of electro-conductivity of the particles [11,35,40,138,153,[158], [159], [160]].

### 4.1 Electrostatic separators

Electrostatic concentrators are the most economic and reliable unit process utilized in the beneficiation of primary minerals like in the processing of beach sand and HM deposits composed of zircon, rutile, ilmenite, garnet, leucoxene, and certain secondary minerals. Electrostatic concentrator, as a unit operation has developed increased demand in the beneficiation and processing of zircon associated HMs as well as secondary minerals as a result of the increase in environmental awareness [40]. According to Ravishankar and Kolla [138], various types of electrostatic concentrators exist, subject to charging mechanisms. Thus, there are different types of electrostatic concentrators, which include but not limited to the high/low tension roll (HTR/LTR) electrostatic separators, electrostatic plate (ESP) separators, electrostatic screen static-field separators (ESS), triboelectric or tribostatic separators, etc. Howbeit, the conventional or the most commonly used electrostatic concentration employed in mineral sands or beach sands, alluvial mineral deposits and HM operations involves the use of the combination or either of electrostatic plate (ESP) separators and high tension roll (HTR) electrostatic separators [30,138,141,145,[161], [162], [163]]. However, triboelectric or tribostatic separators have in recent times developed certain interest worldwide, and have been widely investigated by numerous researchers. For instance, the solid HMs that are being processed by triboelectric separators may be in any combination form of mineral particles such as conductors, semi-conductors and non-conductors. Hence, the triboelectric separators are being considered to have a more wide potential than the HTR (corona) separation in the sense that only the conducting mineral particles (conductors) are being separated from the non-conducting mineral particles (non-conductors) [154,[164], [165], [166], [167], [168], [169]].

It has also been reported that a triboelectric/tribostatic separator makes use of electrodes that are insulated in order to curtail the attraction of materials or mineral particles to it from the loss of its charge on contact. In addition, the tribostatic separator was reported to give higher separation efficiencies and better effects when dealing with reduced or finer sizes of HM particles. Hence, the tribostatic separator was reported to possess certain merits over other electrostatic separators; which include the potential to process or concentrate fine-grained mineral deposits and thus, reduce the number of process steps or stages involved in the mineral beneficiation as well as the physical units involved in the beneficiation flow sheet [30,170]. However, this would require certain modifications in order to control the possible emissions of dust particles as a result of the high levels or measure of radionuclide composed in the fine-grained zircon mineral [30]. Its applications are however limited to the mineral species that are to be contact charged in order to be separated from the non-conducting mineral species [138]. Often times in electrostatic separators, there is a generation of substantial streams of middlings composed of conductors as well as non-conductors. These middlings then would require a re-circulation back to the early stages of the system or circuit. This procedure however resulted to increased number of process steps or stages. Howbeit, there have been several reports on the modifications to this process by various researchers [30,162,170]. This method of mineral processing has been established an effective beneficiation process by several researchers in recovering and separating Zr minerals; as Zr minerals are mostly non-conductive and are collected amongst the non-conducting mineral portions during electrostatic concentrations. Although, multiple stages electrostatic concentrations is recommended for optimal recovery and efficiency; since there is also an establishment that certain amounts of some other non-conductive gangue minerals like the aluminum silicates, monazite, kyanites, and quartz may also be captured in the non-conducting mineral portions [30]. Finally, there have been a lot of different varieties of electrostatic concentrators (which includes ionized field and induced charge separators) have been produced or invented over the last century. Howbeit, there are only but a few fundamental changes in the commercial/industrial designs of electrostatic separators [138].

### 4.2 General principles and influencing factors of electrostatic concentration

The basic principles of electrostatic concentration have been established over the decades. It is worthy to note that before electrostatic concentration can be feasibly applied, certain prerequisites are to be considered and attained. The behavior of mineral particles is however dependent on their geometrical and electrical characteristics. Thus, electrostatic concentration of mineral particles can be feasible when the ambient conditions, separation unit/system, and electric field variables/parameters are being applied appropriately [137]. Basically, there are four groups of factors to be considered that influence the feasibility of electrostatic concentration; which are: the environment and ambient conditions, equipment and design, mineral properties and characteristics as well as that of the powder particles [171]. Therefore, success of electrostatic concentration or its outcome is highly dependent on specific process conditions, factors, variables or parameters, such as: the dielectric constant and conductivity of the mineral particles, electric field strength, shape and PSD of the mineral particles as well as the mineralogy or composition of the minerals or materials that is involved in the beneficiation or concentration process [137,139,[141], [142], [143], [144], [145],[171], [172], [173]]. Also, there may be certain other influencing factors which may depend on the equipment type of the electrostatic concentration. On this note, the mineral to be separated should be comprised of individual mineral particles or grain sizes, where comminution and reduction of particle grain size is essential in order to acquire uniform mineral types, so that liberation can occur [137,[174], [175], [176]].

**Fig. 13:**
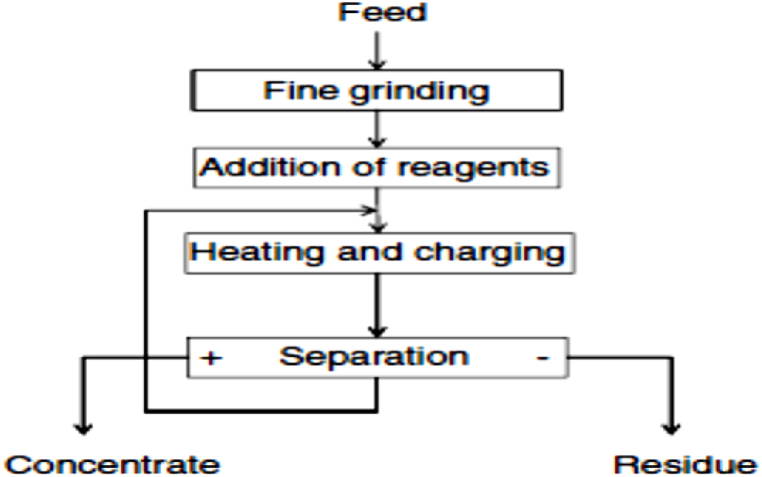
Flow diagram of a typical electrostatic concentration circuit [137].

However, potential or voltage intensity, roll or rotational speed, temperature, feed rate and electrode configuration are other vital parameters and influencing process applications, mineral and atmospheric factors or conditions that needs to be considered for optimal electrostatic concentration performance of the minerals [139,[141], [142], [143], [144], [145],^172^,^173^]. Also, this process of physical separation or concentration is influenced by certain forces that act on the mineral particles that are to be beneficiated. These forces include: electrostatic force, van der Waals and gravitational forces [137]. In addition, there are several existing reports on the influence, interplay determination and also on the operational, process optimization and process modeling factors of these particular forces in their various types of electric force fields on the trajectory of the mineral particles that is being concentrated [137,139,173,[177], [178], [179], [180], [181], [182], [183], [184], [185], [186]]. It is also worthy to note that a prior heating of the mineral particles in a heating oven or on a hot plate for up to 100–150 °C before the electrostatic separation enhances the separation efficiency of the zircon or Zr mineral from other heavy minerals (HMs). However, electrostatic concentration process is made up of two basic steps, which includes the charging and the separation process. Fig. 13 shows the flow diagram of a typical electrostatic concentration circuit of HMs.

### 4.3 Charging

Charging is regarded as a very key factor involved in the electrostatic concentration of HMs as a result of its very high influence on electrostatic separation processes [137]. The technology in electrostatic concentration, more especially the triboelectric separator greatly relies on the charging of the mineral particles before they are being exposed to the electric fields. There are several process ways where the charging of minerals or materials may be effected. Howbeit, there are three primary charging methods that are practical commercially. These charging methods include: triboelectrification (tribocharge or friction), ion bombardment (also referred to as corona discharge) and induction [137,[142], [143], [144],^154^,^187^]. The induction and ion bombardment or corona discharge mechanisms tend to exploit the differences in conductivity of the different HMs. Howbeit, all three mechanisms involve the transfer of charge to/from mineral particles [137]. The tribocharging or triboelectrification can be caused by the particle-surface and/or particle-particle collisions. These collisions may be generated by fluidized beds or vibrations and by mechanical or gas conveyance [154]. Albeit, tribocharging/trieletrification is a somewhat complicated process, the charge sign and magnitude established on the mineral particles is dependent on the workfunction difference or the level of Fermi energy of the two materials in contact with each other [144,154,188]. More so, the net charge or magnitude on mineral particles is also dependent on the crystal orientation and PSD of the minerals, and as well, it increases generally with the velocity of conveyance as well as the PSD [154,189]. Also, the relative humidity has been reported by researchers to show somewhat complex effects on the mineral particle charge [154,169,190]. According to Jiang et al. [154] and Bennett et al. [191], it was observed that the triboelectrification process may be reduced by means of the fine mineral particles addition during transportation of certain mixed lactose powders to a stainless cyclone.

Charging of the mineral particles occurs majorly on the surface of the particles. Thus, the influencing factors of charging are surface-based factors. The main essential physical property is the surface conductivity of the mineral particles. In that regard, a high surface conductivity value tends to favour charging of the mineral particle. Howbeit, increase in the surface conductivity will easily result to a more rapid discharge of a previously charged mineral particle. Considerations are to be taken on the feasibility of the minerals conductivity to be successfully utilized for electrostatic concentrations [137]. In furtherance, surface work function of a mineral is essential for charging (more especially on triboelectrification charging). Also, the polarity as well as the maximum potential between particles of two minerals to be separated can however be determined by the surface work function differences [137,[192], [193], [194], [195]]. In addition, Dötterl et al. [137] reported that the surface work function and the conductivity properties of a particular solid mineral surface are regarded not unique characteristics of the particular HM. Hence, they greatly depend on the ambient conditions like: temperature, moisture, chemicals, pressure, and also the size and purity of the HMs. Also, the mineral particles’ shape influences how the surface of mineral particles is covered by absorbents during the surface conditioning. Howbeit, the absorbent behavior changes on the mineral surface may result to differences in the surface work function and surface conductivity. This is as a result of their influence on the chemical composition and structure of the mineral particle surface [137]. More so, under ambient conditions, a mineral particle surface may bear at least a layer of absorbed molecules which is usually water. The water absorbed may lead to the very rapid discharge of the previously charged mineral particles. Thus, a heating process prior to an electrostatic concentration process is imperative in most scenarios. At the temperatures, between 100 and 150 °C, only just the physically absorbed water is eliminated. However, the heating process to a temperature >150 °C is usually regarded as being not economical and will also tend to hinder sufficient charging. In conclusion therefore, these factors are termed of great influence to electrostatic concentration charging (especially the triboelectrification charging) [137].

#### 4.3.1 Charging by (conductive) induction

**Fig. 14:**
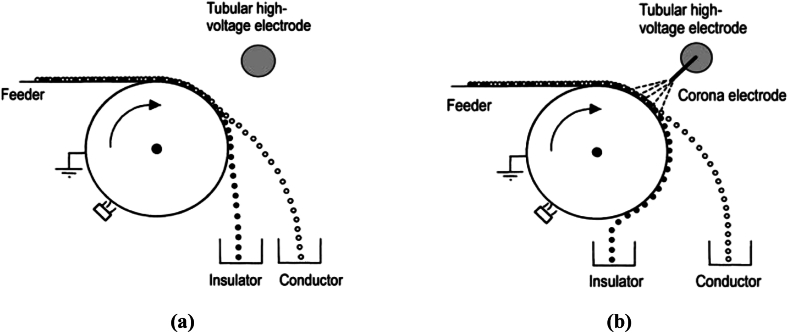
Rotational drum electrostatic concentrator via (a) inductive (b) corona charging [137].

This type of charging usually favours the separation of the conducting minerals (conductors). The mineral feed to be separated is fed to a grounded rotational drum in order to pass through an electric field that is generated by a tabular high voltage electrode. In most scenarios, the electrode is usually negatively charged, however positive charging is also possible, regardless [137]. The conducting mineral particles are therefore, charged by induction within a short period of time or interval. This is however dependent on the surface conductivity of the mineral particles [137,[196], [197], [198]]. The induced charge however has an inverse polarity to that of the high voltage electrode. Thus, attractive forces are imposed on the conducting mineral particles and then leave the rotor towards the electrode. This will then lead to a stream of deflected mineral particles from the rotor surface and can then be subsequently collected in a well-positioned container. Also, the non-conducting mineral particles are not charged significantly during their rest on the rotor or it may be that their charges are very low to actually effect the movement towards the high voltage electrode [137]. Therefore, they tend to remain on the rotor until they are scraped off by a brush or force of gravity allows them fall down into their collecting container. However, this process reportedly, doesn't have a high efficiency. This is because all the conducting mineral particles must have a contact on the rotor for an induced charge to be transferred made feasible. Hence, there is the recommendation of single layer mineral particles in order to obtain proper mineral separation. The equipment utilized for this electrostatic concentration via inductive charging is the drum electrostatic concentrator. This equipment however can just be utilized for separating the conducting mineral particles from the non-conductors. Albeit, the separation of mineral particles that have high surface conductivity differences have not been entirely successful or completely employed on a large scale. The drum electrostatic concentrators are particularly employed during the final cleaning of industrial HM products like zircon, rutile, etc. [137]. Fig. 14a and 14b shows rotational drum electrostatic concentrator via inductive and corona charging, respectively.

#### 4.3.2 Charging by ion bombardment or corona discharge

**Fig. 15:**
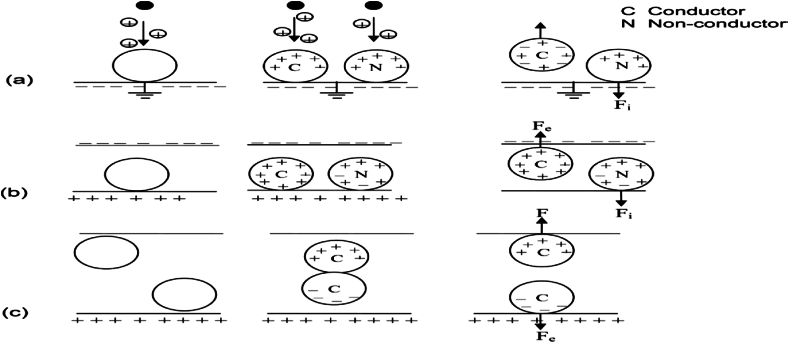
Mineral particle-charging process (a) Charging by corona (b & c) Charging by conductive induction [39,143].

The electrostatic concentration via ion bombardment or corona discharge functions similarly to that of the inductive charging process. Contrary to inductive charging process, both the conducting and non-conducting mineral particles are charged as a result of high electrical field strength or tension which is produced by ionizing corona electrode that generates an ionic stream. Thus, the corona electrode often comprises of a needle-like or thin wire electrode. The charge of the semi-conducting and conducting mineral particles then migrates rapidly to the grounded rotor. Then, the non-conducting mineral particles will remain however partially charged for a longer period of time as a result of their greater surface resistivity. Thus, they are attracted to the rotor, making them migrate around the surface and they are being scraped off with a brush and collected in a container. This type of electrostatic concentration is often referred to as corona separation and it could be employed for separating HM particles of different conductivities. In conclusion therefore, this corona separation process is in contrast to the drum electrostatic concentration as the corona separators tend to provide a more wide applicability range as a result of their stronger/higher field that is employed [137]. Fig. 15a, 15b and 15c respectively shows mineral particle-charging process of corona and conductive induction charge.

#### 4.3.3 Charging by friction or triboelectrification or tribocharge

**Fig. 16:**
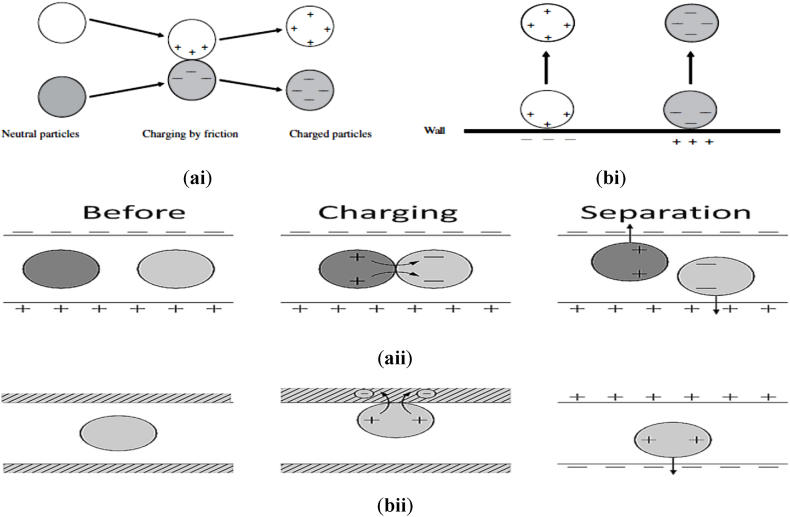
Triboelectric charging involving (a) Contact between mineral particles (particle-particle) (b) Contact between mineral particles and wall of charger [137,171].

This type of charging usually favours the separation of the non-conducting or insulating minerals [137]. Triboelectrostatic concentrators have been used successfully to separate two non-conducting minerals, such as: zircon, quartz, monazite, kyanite, staurolite, sillimanite, garnet and also in the separation of minerals and plastics [40]. Two different minerals are charged upon friction against each other is a well understood phenomena; and this is seen as the physical basis of the charging by friction or triboelectrification or triboelectric charge or tribocharge. This is however in contrast to the rotational drum electrostatic concentrators as in the sense that the charging as well as subsequent separation process are more distinct processes. The charging of mineral particles may occur on contact with other particles within the mineral ore/deposit or in contact with the charging device material [137]. This is however displayed in Fig. 16. It was reported by Lacks [199] that the triboelectric charging phenomena have not been completely investigated. The researcher reported the difficulty of prediction due to the variety and complexity of factors of which could influence/affect the charging process. More so, a lot of factors could influence and affect the outcome achieved by the triboelectric charging process. This phenomenon is reported unwanted in a lot of applications because the electrostatic discharges that results may damage the electronic equipment or can further initiate explosions [137,199]. Hence, lots of research study has been carried out in order to mitigate the triboelectric charging [200,201]. Nevertheless, the selective mineral particle triboelectrification has been reported as a key step process as well as a prerequisite for a successful electrostatic concentration process [137,150,195,202,203]. Therefore, Fig. 16 shows the triboelectric charging of mineral particles. Table 4 enumerates the influential factors to be considered during triboelectrostatic concentration.

**Table 4:** Factors to be considered during triboelectrostatic concentration

Factors	Details	References
Environmental conditions & influence	Influencing parameters on the charge behavior of mineral particles, such as: temperature, humidity, light & atmosphere	Mirkowska et al. [171], Greason [192], Ndama et al. [204], Ireland and Nicholson [205], Baytekin et al. [206], Manouchehri [207]
Powder particle properties (Behaviour of particle systems)	Property differences influences the contact/contact stress type: powder composition (different mineral ratios), particle shape differences, Mean particle size & PSD, multiple faceted materials, polycrystalinity	Mirkowska et al. [171], Manouchehri et al. [187,208], Kronik and Shapira [209]
	Behaviour of other body systems influences the system's forces & contacts: charge decay in separation, agglomeration of particles, particle surface non-uniform distribution of charge, laminar flow disturbance of particles, separation zone contacts, behavior difference in blunt & sharp (edges & flat) planes	Dötterl et al. [137], Mirkowska et al. [171], Kronik and Shapira [209], Ireland [210], Matsuyama and Yamamoto [211]
Mineral/material properties (basic physicochemical characteristics)	Surface properties influence contact type & transport of charge: roughness of surface, plane orientation, charged surfaces of new cleavage, electrical properties (which includes surface conductivity & resistivity, surface states, effective work function, electric permittivity), surface contamination & functionalization (presence of water layer on surface, adsorbed atoms & ions, chemical conditioning), dopant & impurities, termination of surface	Mirkowska et al. [171], Manouchehri et al. [187], Ndama et al. [204], Kronik and Shapira [209], Melitz et al. [212], Matsusaka et al. [213], Brillson [214], Dwari et al. [215], McCarty and Whitesides [216], Stachowiak and Batchelor [217], Lowell and Rose-Innes [218], Nordhage and Baeckstroem [219], Carta et al. [220]
	Exchange of charge & electronic structure is determined by the bulk properties: crystallographic structure, chemical composition, terminal crystallographic planes' orientation, dopants & impurities, conductivity & resistivity, work function value, mechanical property difference	Mirkowska et al. [171], Manouchehri et al. [187], Ndama et al. [204], Dwari et al. [215], Nordhage and Baeckstroem [219], Carta et al. [220], Bocquet et al. [221], Persson [222], Ciccu et al. [223]
Equipment & equipment design influence	Actions before separation influences charging behaviour: chamber conditioning (drying, heating, chemicals introduction on surfaces of the mineral particles), aging, contamination, precharging, transporting, milling, storing	Mirkowska et al. [171], Carta et al. [224], Matsusaka et al. [225]
	Parameters of separation influences the system forces: electric field magnitude & distance btw electrodes, electrode type (rotating plate), electrodes modification & isolation	Dötterl et al. [137], Mirkowska et al. [171], Billici et al. [226], Masuda et al. [227]
	Parameters of charging influences charge exchange value, contact stress, contact type, No of contacts: time btw separation and charging, rate of feeding (throughput), charger unit type & parameters (plate charger vibration frequency, the belt travel speed), charger wall material & the electrical properties	Dötterl et al. [137], Mirkowska et al. [171], Manouchehri et al. [187], Ndama et al. [204], Kronik and Shapira [209], Dwari et al. [215], Ciccu et al. [223], Kwetkus and Sattier [228]

### 4.4 Triboelectric series and transfer of charge mechanisms

The charge polarity that a particular mineral or material acquires upon friction is dependent on the surface work function difference of the minerals to be separated. Thus, certain minerals have been arranged in series and it is being arranged according to the charge polarity received by the process of friction. This however is referred to as the triboelectric series [137]. Howbeit, a lot of researchers have made the triboelectric series a case study as a result of its lack of uniqueness whereby a mineral or material occupying a particular position. Thus, several triboelectric series of different materials have been reported in the open literature [137,[229], [230], [231], [232], [233], [234]]. This triboelectric series may be utilized in the preliminary selecting of charger materials against the mixture or combination of mineral particles (McCarty and Whitesides, 2008; Dötterl et al., 2016). Table 5 shows the triboelectric series of certain minerals with the minerals from up to down preferring positive charging or the low surface work function while the minerals from down to up prefers the negative charging or high surface work function.

**Table 5:** Triboelectric series of certain minerals [137,232].

↓ Positive charging (Low surface work function) ↓
Apatite
Carbonates
Monazite
Titano-magnetite
Ilmenite
Rutile
Leucoxene
Magnetite/Hematite
Spinels
Gamet
Staurolite
Altered Ilmenite
Geothite
Zircon
Epidote
Tremolite
Hydrous Silicates
Alumino-silicates
Tourmaline
Actinolite
Pyroxene
Titanite
Feldspar
Quartz
↑ **Negative charging (High surface work function)** ↑

### 4.5 Charging contact between metals and insulators

Triboelectric charging occurs on contact between metals (conductors) and the charges generated easily and rapidly dissipate or decay [137,235]. However, when a metal is in contact with a non-metal (insulator), the outcome is somewhat different. In contrast to the situation where the charge on a metal rapidly dissipates, insulators are being charged and can also retain the charge on contact with metals [40,137,210,[236], [237], [238], [239], [240], [241], [242], [243]]. The exploitation of the triboelectric charging between metals and insulators in order to influence the triboelectric charging process of mineral particle mixtures has been carried out, such as the modifying of the tribocharging wall material [137,227]. The surface work function or the metal position must be selected in such a way that it lies between the minerals or materials to be separated. The metals or insulators relative position is not constant; however it is highly dependent on temperature [137,194,244].

### 4.6 Challenges involved in electrostatic concentration

There have also been several challenges encountered over the years, with the use of electrostatic concentrations in the processing of minerals. Although, this process of concentration has been reported effective in the beneficiation of certain HMs such as zircon [30], it is however regarded (reportedly) not very efficient as a result of some certain shortcomings/demerits. There have been reports of low separation efficiency with electrostatic concentrations. This is chiefly due to the fluctuations in ambient conditions. Such conditions include: humidity or moisture, as well as temperature of the mineral feeds [138,173,245].

#### 4.6.1 Ambient conditions: influence of temperature, humidity and pressure

Increased humidity tend to decrease the separation efficiency of electrostatic concentration process; which is as a result of the indiscriminate condensation of the conducting layer of water on the minerals and thereby results to the misreport of the non-conducting minerals on to the conducting minerals. For instance, Mohanta et al. [246] reported that humidity or moisture has significant influence on the charge accumulation magnitude of the mineral particles. The authors in a certain study, reported that as the relative humidity increases, charge accumulation magnitude is decreased and hence, the establishment that humidity has significant influence or effects on the separation efficiency of electrostatic concentrations, especially on the triboelectrostatic concentration. Therefore, better electrostatic concentration results are usually achieved when the humidity is low. Howbeit, the process factor which is more economical in lowering the humidity at certain particular ambient conditions is the increase in temperature. Since, according to the reports by several researchers, temperature change is linked directly to relative humidity alterations, and as such, humidity as well as temperature contributes significantly in influencing triboelectric charging; and also, the dehumidifying of large air amounts is somewhat expensive [137,193,206,[247], [248], [249]]. Therefore, temperature is reported to have major influences on the conductivity of HMs as well as the separation efficiency of electrostatic concentrations [137,138,141,246].

It is also worthy to note that dew point may be reached on the surface of the mineral particles when relative humidity is very high. HM ores that have been exposed to high relative humidity must undergo a thorough thermal treatment so that the excess water absorbed can be evaporated. Thus, the mineralogy, type and history of a particular HM deposit may be a determinant factor in the outcome of (tribo)electrostatic concentrations [137,224]. On the other hand, as too much water hinders triboelectric charging, low separation efficiencies could also be achieved for certain minerals in the absence of water. Like in the case of potash mineral ores where water adsorption on magnesium sulphate or alkali metal chloride surfaces is vital for selective tribocharging of the potash mineral ore [137,[250], [251], [252], [253], [254], [255], [256], [257]]. It was further reported and discussed that the triboelectric charges dissipate rapidly in low pressures [137,[258], [259], [260], [261]]. More so, several charge transfer models have shown the usefulness of thin water layers during the process of triboelectrification [137,229,262].

#### 4.6.2 Chemical conditions

Apart from ambient conditions, some certain chemical conditions could affect the actually efficiency of triboelectric charging. Despite the fact that certain undesired chemical contaminations could reduce the efficiency of electrostatic concentrations [137,263], addition of certain conditioning chemical agents could enhance the electrostatic concentration of HMs as well as other materials [137,244,[264], [265], [266]]. Thus, there would be little or no selectivity without the additions of such chemical agents. For instance, in the triboelectric concentration of some certain HMs, certain chemical reagents or conditioning are regarded as prerequisites in order to achieve a high yield cost effective mineral concentration. The adsorption of some organic substances on the mineral particle surfaces could affect its triboelectric charge properties [137,[267], [268], [269], [270], [271], [272], [273]]. Also, a huge difference exists when the chemical compounds or agents are utilized as bases, acids or neutral salts, hence, the proper selection and application of different chemical reagents and conditions for separation efficiency of the (triboelectric) electrostatic concentration process [137]. Despite that a whole lot of investigations have been conducted in that regard, there is still a lack of complete understanding regardless, for the process of triboelectric charging of chemically conditioned mineral particles. Thus, the design of equipment still requires further or extensive pilot plant and lab test investigations.

**Fig. 17:**
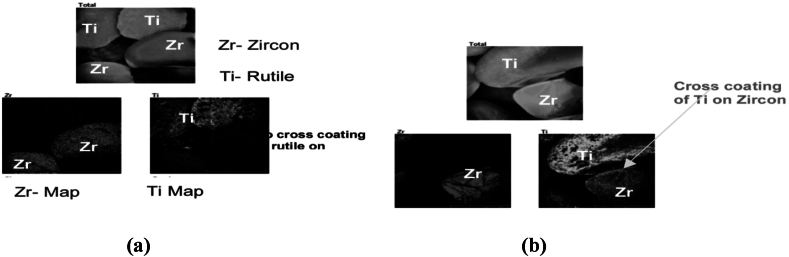
Cross contamination affecting separation efficiency **(a)** good separation efficiency **(b)** low separation efficiency [138].

**Fig. 18:**
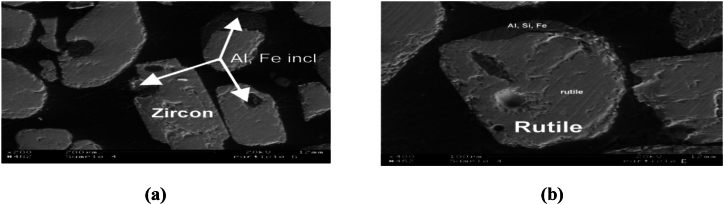
Surface characterization of heavy feed minerals with inclusions of impurities **(a)** Zircon mineral **(b)** rutile mineral [138].

In furtherance, according to Ravishankar and Kolla [138], average separation efficiencies of approximately 70 % results to substantial load recirculation. It was however reported that in a plant having a feed rate of 50 tons per hour, even a 1 % improved separation efficiency will improve the production rate of the mineral product by at least 3 %. Also, besides the process conditions, a major shortcoming/challenge involved in electrostatic concentration process is the surface contamination or impurities. For instance, Fig. 17a and 17b illustrates HM feeds composed of zircon and rutile minerals, which are with/without cross contaminations. The separation efficiency loss as a result of the misreporting of the minerals such as: the non-conductors (zircon mineral) with Ti mineral species coating to conducting fractions, thereby resulting to a low mineral grade quality of the final mineral product. There are other types of contaminations, such as: the indiscriminate coating of clay and other species like Fe, Ti and Al; which also results to a reduction in the separation efficiency of the concentration process [138]. Fig. 18a and 18b illustrates Al and Fe silicates as inclusions in zircon mineral; and Al and Fe silicates as impurity coatings on the rutile mineral.

### 4.7 Electrostatic separation enhancer technology

Ravishankar and Kolla [138] also investigated and reported the use of a technology of electrostatic chemical enhancers. This study was carried out in order to improve the separation efficiency of HMs such as zircon and rutile minerals. The researchers obtained chemical enhancement of electrostatic separation through the judicious combining of selective surface chemical reagents which modify/favour surface conductivities for achieving enhanced separation efficiencies. For instance, an enhancement/increment of about 8–11 % in the separation efficiencies of zircon and rutile was obtained by Ravishankar and Kolla [138] during their first pass on a lab high tension electrostatic separation. The authors in conclusion, reported that the chemical enhancer technology: (i) is somewhat robust (ii) is very easy in handling and usage as well as in training of operators (iii) could be expanded to a technological platform and could be potentially utilized in separating chemically dissimilar conducting minerals as well as the non-conductors (iv) could enhance production rate as a result of the load recirculation reduction (v) is somewhat adaptable to the conventional electrostatic concentration with minimum investment of capital (vi) could translate into saving of energy by eliminating a number of cleaning phases. However, further study on the electrostatic chemical enhancers/technology is still ongoing as regards to process the variables or plant parameters. In conclusion therefore, Table 6 depicts a concise summary of electrostatic concentration of HMs deduced from literature.

**Table 6:** Summary of electrostatic concentration of HMs deduced from literature

Reference	Heavy mineral	Electrostatic concentrator
Sampaio et al. [88]	Zircon mineral concentrates	Corona electrostatic separator
Aral et al. [89]	High grade zircon-rich associated HM concentrates	Corona electrostatic separator
Knoll and Taylor [152]	Zircon associated rutile mineral	ESP & HTR separators
Rejith and Sundararajan [158]	Beach sand deposits	ESP & HTR separators
Lawver and Hopstock [163]	Zircon associated tungsten & tin minerals	HTR separator
Dance and Morrison [173]	Mineral sand deposits	ESP & HTR separators
Lawver and Dyrenforth [274]	Beach sand deposits	HTR separator
Venter et al. [275]	Zircon and rutile mineral concentrates	Corona electrostatic separator

## 5 Conclusions

Magnetic and electrostatic techniques in the beneficiation of HMs have been reviewed and discussed accordingly, concisely described and also in simplified comparisons. From the descriptions made in the published literature, it is evident that a high demand exists in the adoption of simple, cost effective and eco-friendly physical beneficiation routes for concentrating certain HMs. This includes the ferromagnetic, paramagnetic, diamagnetic, conducting and non-conducting HM particles. On this premise, it is therefore, noteworthy that the choice or selection of physical beneficiation techniques of HMs is heavily dependent on mineralogy, composition, shape, PSD and the physicochemical properties or characteristics of the HMs. Both magnetic and electrostatic concentrations have significant effects in the concentration and upgrade of HMs for the purpose of successful, less complex downstream pyro-/hydrometallurgical extraction procedures for the recovery of the values. Nonetheless, the performance efficiency of magnetic and electrostatic concentrators is highly dependent on certain process conditions, such as the strength of magnetic or electrostatic field of the concentrators, mineral's response or susceptibility level to magnetism and electrical conductivity, mineralogical distribution, process and concentrator variables, concentrator throughput, PSD of feed particles and magnetic or electrostatic distribution characteristics possessed by such HM particles.

The main difference between the HIMS techniques discussed in this study is simply based on their capacity and separation efficiency. Among these physical techniques, are the IRMS and RERMS, which are often utilized as rougher and cleaner process of larger capacities. However, the CBMS and LRMS may be employed at the cleaner stages as a result of the low handling capacity. The RERMS has been reported a cost efficient unit system amongst the other concentrators/techniques as a result of the high throughput and low energy consumption. In addition, between the IRMS and RERMS techniques, the IRMS possess the demerit of being an electro-magnetic concentrator type, which significantly influences the operating cost of the concentration process. It also possesses flexibility in adjusting the magnetic field strength/intensity which is simply on the basis of the change in the mineral feed properties/characteristics. More so, on the other end, the RERMS may be utilized in the upgrading process of coarse heavy HM particle size fractions of between 75 μm and 10 mm. Howbeit, the concentration of close range of particle sizes is more effective in these concentrator types, which tend to minimize the entrainment of finer mineral particles of lower magnetic susceptibility to the magnetic stream. Therefore, a magnetic concentration system circuit comprised of the combination of these concentrators may assist in the separation efficiency as well as in the economics of the concentration process of specific complex paramagnetic HMs.

In addition, the triboelectrification or tribocharge phenomena during triboelectrostatic concentrator have been briefly reviewed. For good separation efficiencies, the adjustment of certain parameters or variables of the concentration system is imperative. Certain mineral properties such as electrical and mechanical properties of the mineral particles, the charging unit properties, the concentrator type and the conditions of the environment are to be considered. Contact charging and its effects on mineral solids should be attributed special attention as the charge generation and charge conservation on the mineral particles is very vital and influential for effective electrostatic concentration process. In the quest for a cost effective and an eco-friendly concentration technique, indebt knowledge and understanding of the general physical mechanisms is imperative and hence, further investigations and advanced research is required on the somewhat complex triboelectric or tribocharging process for future enhancements in the electrostatic concentration of HM particles.

## Recommendations

The efficiency of the aforementioned physical beneficiation processes on HMs has been investigated. The feasibility of efficient physical concentrators and separation techniques on HMs often meets certain challenges. Hence, proper mineral characterization and exploitation of the mineral's physicochemical characteristics/properties as well as the exploration and optimization of various process techniques are highly imperative. This is not far from developing suitable mineral characterization strategy in order to comprehend the magnetic and electrical properties or characteristics distributed among the mineral particles of the HM deposits. Currently, there is need for developing and enhancing efficient mineral processing routes of dry and wet HIMS (more especially the paramagnetic concentrators) as well as the corona and triboelectric or tribostatic electrostatic separators for the recovery of fine paramagnetic as well as (semi)conducting/non-conducting low-grade HM deposits. Future research should therefore, be focused on the inter-twined effect analyses of PSD, density and SG, and their magnetic susceptibility and electrical conductivity responses as well as their behavior in magnetic/electrostatic force fields with certain operating and designing parameters/variables of both processes and concentrators. In addition therefore, their applications in the concentration of various (para-) magnetic and electrical conducting minerals with very complex mineralogy require full exploration. More so, the shortcomings existing in the use of HIMS for magnetic concentration and HTR electrostatic concentrators may be resolved if the science behind the movement of mineral particles or the particle motion physics involved in magnetic and electrostatic concentration is completely understood as well as the distribution of the magnetic susceptibility and electrical conductivity responses of the feed mineral deposit.

Lastly, a comprehensive optimization process of the selected physical mineral processing or beneficiation technique is imperative, applying varying and differing parameters or variables in order to obtain optimum beneficiation conditions involved in the physical processing of HMs as well as equipment type consideration. Thus, in order to achieve optimum results in the HM upgrade and concentration, high separation efficiencies, grade purities and mineral recoveries, the efficient optimization and control of process parameters and/or variables or the system conditions involved in the adopted HM physicochemical beneficiation process route is therefore, highly imperative and greatly recommended. Hence, this can however be obtained with Design Expert software or with Taguchi-Anova design and model, by proper experimental design process of the involved concentration procedures, which should also include suitable experimental process optimizations as well as comprehensive engineering models and simulation mechanisms of various aspects of such physical concentration and beneficiation procedures.

## Ethical approval

There are no direct or indirect ethical issues with respect to this research paper.

## Consent to participate and publish

The authors have consented to participation in the drafting and publication of this manuscript. Hence, the processing of this manuscript can proceed without any concern of dispute between direct and indirect parties involved.

## Funding

There was no funding accredited to this research work.

## Availability of materials and data

The data and materials presented in this article are available from the authors upon request.

## CRediT authorship contribution statement

**Nnaemeka Stanislaus Nzeh:** Writing – original draft, Visualization, Software, Resources, Investigation, Data curation, Conceptualization. **Abimbola Patricia Popoola:** Writing – review & editing, Visualization, Validation, Supervision, Project administration, Funding acquisition, Formal analysis.

## Declaration of competing interest

The authors declare that they have no known competing financial interests or personal relationships that could have appeared to influence the work reported in this paper.
